# Communication and deniability: Moral and epistemic reactions to denials

**DOI:** 10.3389/fpsyg.2022.1073213

**Published:** 2023-01-04

**Authors:** Francesca Bonalumi, Feride Belma Bumin, Thom Scott-Phillips, Christophe Heintz

**Affiliations:** ^1^Department of Cognitive Science, Central European University, Vienna, Austria; ^2^Department of Economics and Business, Central European University, Vienna, Austria; ^3^Institute for Logic Cognition Language and Information, University of the Basque Country, San Sebastian, Spain

**Keywords:** deniability, Relevance Theory, strategic speaker, indirect communication, pragmatics, accountability

## Abstract

People often deny having meant what the audience understood. Such denials occur in both interpersonal and institutional contexts, such as in political discourse, the interpretation of laws and the perception of lies. In practice, denials have a wide range of possible effects on the audience, such as conversational repair, reinterpretation of the original utterance, moral judgements about the speaker, and rejection of the denial. When are these different reactions triggered? What factors make denials credible? There are surprisingly few experimental studies directly targeting such questions. Here, we present two pre-registered experiments focusing on (i) the speaker’s incentives to mislead their audience, and (ii) the impact of speaker denials on audiences’ moral and epistemic assessments of what has been said. We find that the extent to which speakers are judged responsible for the audience’s interpretations is modulated by their (the speakers’) incentives to mislead, but not by denials themselves. We also find that people are more willing than we expected to revise their interpretation of the speaker’s utterance when they learn that the ascribed meaning is false, regardless of whether the speaker is known to have had incentives to deceive their audience. In general, these findings are consistent with the idea that communicators are held responsible for the cognitive effects they trigger in their audience; rather than being responsible for, more narrowly, only the effects of what was “literally” said. In light of our findings, we present a new, cognitive analysis of how audiences react to denials, drawing in particular on the Relevance Theory approach to communication. We distinguish in particular: (a) the spontaneous and intuitive re-interpretation of the original utterance in light of a denial; (b) the attribution of responsibility to the speaker for the cognitive effects of what is communicated; and (c) the reflective attribution of a particular intention to the speaker, which include argumentative considerations, higher-order deniability, and reputational concerns. Existing experimental work, including our own, aims mostly at (a) and (b), and does not adequately control for (c). Deeper understanding of what can be credibly denied will be hindered unless and until this methodological problem is resolved.

## 1 Introduction

There is always a gap, however small, between what is linguistically encoded in a sentence and what is communicated by the speaker when using that sentence in context (Carston, 2004)—as Grice (1989) famously put, between “what is said” and “what is meant”. As a consequence, it is always possible in theory to deny that what an audience has inferred was indeed what was actually meant. This in turn raises the prospect that deniability could be used in a strategic way, such that speakers generate indirect formulations when there is a risk that their intended meaning may cause an undesired response (Brown and Levinson, 1987; Pinker, 2007; Pinker et al., 2008; Lee and Pinker, 2010). Classic examples include acts of bribery (“I’d do that for anybody who needs a proper guidance.”), sexual innuendos (“It’s going to be a long night. […] And I don’t particularly like the book I’ve started.”) and insinuations (“Handsome armour. Not a scratch on it.”).^1^ In practice, of course, some denials of intended meaning are far more credible than others. These possibilities raise important questions about commitment and credibility in language use. What factors make denials credible? What kind of cognitive reactions do audiences have to different types of denial? Such questions are important not only because answering them would shed light on the cognitive processes involved in communication; but also because denials and strategic use of language have a pervasive role in our daily life, and its consequent influence in domains such as politics and the law.

There are few experimental studies that directly target these issues, despite the important role that denials and deniability play in human interaction. Most of previous research on deniability and implicit communication consists of theoretical contributions, stemming from a widely spread assumption that denying what was implied should be more credible than denying what was explicitly expressed (Fricker, 2012; Camp, 2018). Deniability has been also taken to jeopardise the necessary public responsibilities ensuring that testimony provides reasons to believe the transmitted knowledge (Peet, 2015; Davies, 2019). Furthermore, deniability has been examined as a type of defence strategy that a speaker can appeal to in order to deny their commitment to an ascribed meaning (i.e., meaning initially ascribed by the audience) (Boogaart et al., 2020; see also Morency et al., 2008). Looking at the experimental literature, instead, there is, to our knowledge, little research targeting these research questions (for exceptions see Sternau et al., 2015; Reins and Wiegmann, 2021; Bonalumi et al., 2022). The most developed line of experimental research in this area targets not audience interpretations, but rather the specific situational conditions when it may be advantageous for speakers to exploit the possibility of denial for their own strategic ends (Lee and Pinker, 2010). By contrast, we know relatively little about the audience side. What cognitive reactions are triggered when a communicator denies having intended the meaning that the audience appears to have actually inferred? When are they triggered, and why?

Given the relative dearth of studies directly targeting audience reactions to denials, here we present two pre-registered experiments targeting the question of what cognitive effects denials can have (§2 and §3). We focus in particular on (i) the role of speaker’s incentives to mislead their audience, and (ii) the impact of speaker’s denials on their audiences’ moral and epistemic reactions. We adopted this particular focus because these are cases where speakers’ incentives to mislead their audience may be relevant factors in determining what audiences might infer. In particular, we reasoned that prior incentives to mislead the audience can provide background information that shapes the interpretation of a denial (Mazzarella, 2021), and thus impacts its credibility. Imagine that you have asked your daughter if she had finished her homework, and she confirms it. After checking what she had to do, you realise that she has not done the math homework due on Friday. When confronting her, she replies “I meant the homework for tomorrow!.” How plausible is your daughter’s denial? We hypothesised that her denial would be deemed less plausible if she had incentives to lie or mislead you.^2^ For instance, she might have been at the same time asking for the permission to go out with her friends. We hypothesised that if the audience is aware of these incentives, this will impact on the credibility of the denial, as measured by the interpretation and re-interpretation of utterances, and moral assessments of the speaker’s communicative behaviour. Our results are partially consistent and partially inconsistent with these predictions. We find that the ascription of the speaker’s responsibility is indeed modulated by their incentives to mislead their audience (§2). However, their denial did not make a difference. We also find that people are more willing than we expected to revise their interpretation of the speaker’s utterance when they learn that the ascribed meaning is false, regardless of whether the speaker is known to have had incentives to deceive their audience (§3). This pattern of results is consistent with the idea that communicators are held responsible for the cognitive effects they trigger in their audiences.

In light of our results, we distinguish three types of cognitive processes that impact on how people react to a denial (§4). These are: (a) the spontaneous and intuitive re-interpretation of the original utterance in light of a denial; (b) the attribution of responsibility to the speaker for the cognitive effects of what is communicated; and (c) the reflective attribution of a particular communicative intention to the speaker, that is based on the evidence that one has to claim that that the speaker lied or intentionally misled their audience.

## 2 Experiment 1: Moral reaction

Experiment 1 was designed to test the hypothesis that the audience holds the speaker responsible for the cognitive effects of their communicative act in view of their (the speaker’s) incentives to mislead the audience. When such incentives are present, the speaker’s denial should be implausible, and as such it should not mitigate their (the speaker’s) responsibility. Here, we operationalised responsibility as the social consequences that the speaker is called to pay in terms of moral blameworthiness.

We reasoned that a mitigation of the speaker’s ascribed responsibility can be considered a reliable proxy for the audience’s acceptance of the denial, and in turn, for the presence of plausible deniability. Thus, we measured the speaker’s ascribed responsibility by asking the participants to rate the speaker’s blameworthiness for misleading the audience. If blame ratings were negatively affected or not affected by the presence of a denial, i.e., if participants would maintain or increase the severity of their blameworthiness judgement in the presence of a denial, that would suggest that such denial was deemed not plausible.

We thus predicted that the speaker’s incentives to mislead the audience would cause an increase in their perceived blameworthiness. We also predicted that the speaker’s denial of the meaning the audience had initially ascribed to the utterance (hereafter, “ascribed meaning”) would lead participants to blame the speaker with less severity if the speaker had no incentive to mislead the audience in the first place.

### 2.1 Methods

The study was pre-registered on Open Science Framework, with sample size, planned analyses and participants exclusion criteria specified. The pre-registration document is available at https://osf.io/jkn57.

#### 2.1.1 Participants

A power analysis that was conducted with RStudio 1.1.463 (R Core Team, 2020) by using the “rsm” package in R (Harrell, 2022) showed that with 500 participants, assuming small to medium effect size, we would obtain approximately 92% of power when α = 0.05. We thus planned to recruit 500 participants *via* Amazon MTurk (Amazon Mechanical Turk^3^), and 11 additional participants were also included in the analysis since they completed the survey before we closed the survey collector. Being above the age of 18 was the only criteria for participant selection. Each participant provided informed consent before the experiment and were paid $0.40 for their participation. Participants who failed the attention check were excluded (*N* = 4), thus the final sample resulted in 507 participants (236 females, 1 other, 1 prefer not to say, M_age = 39): 252 participants were assigned to the incentive condition (125 in the denial condition, 127 in the no-denial condition) and 255 participants were assigned to the no-incentive condition (128 in the denial condition, 127 in the no-denial condition).

The methods used in this and in the following study are in accordance with the international ethical requirements of psychological research and approved by the EPKEB (United Ethical Review Committee for Research in Psychology) in Hungary.

#### 2.1.2 Materials

We created four different scenarios which followed the following structure:

• *Context part* depicted a social situation between a speaker and a listener, and included information about the speaker’s incentives to mislead the audience: in the incentive condition, the speaker was described as having incentives to let their listener believe something, whereas in the no-incentive condition the speaker did not have any incentive or have disincentives to do so.
• *Dialogue part* included the question of the listener about an event; the speaker’s response to the question was an utterance (X) which yielded an ascribed meaning (Y). The dialogue part was identical in all conditions.
• *Negation part* included the information that the listener realises that Y is false. This part was identical in all conditions.
• *Denial part* included the dialogue in which the listener confronts the speaker by stating that “*I thought you said Y*” and the speaker denied having meant that Y (ascribed meaning), and offered an alternative intended meaning (Z) for their utterance X: “*I didn’t say that. I just meant Z*”. Then, the denial part was present only in the denial condition.

As suggested by Mazzarella (2021), we constructed the speakers’ denials as including the offer of an alternative interpretation for the utterance X. Table 1 shows one specific example. All stimuli are available at https://osf.io/bmqk4/.

**TABLE 1:** Example of scenarios structure and measures under four different conditions presented in Experiment 1.

Context/Incentive	Context/No-incentive
Tommy and Thelma have been in a relationship for a few years.	Tommy and Thelma are siblings and have a very close relationship.
They live in the same college dorm. At the beginning of the new term, they meet a new student, Sara, in the dorm cafeteria. Tommy and Sara start spending a lot of time together, and Tommy knows that Thelma also likes Sara and is happy about them hanging out. One day Thelma looks for Tommy and cannot find him anywhere.	
**Dialogue**	
Thelma asks Tommy when he is back: “*Where were you? I couldn’t find you anywhere*.” Tommy answers: “*Sorry, I went to the laundry room.*”	
**Negation** Later, some friends tell Thelma that Tommy and Sara were together that afternoon.	
**Denial**	**No-denial**
Thelma says to Tommy: “*I thought you said that you were doing your laundry.”* Tommy answers: “*Oh no, I didn’t say that. I just meant that I was helping Sara because she didn’t know how to use the washing machine and asked me for help*.”	**x**
**Attention check**	
Who was Thelma looking for that afternoon? • Tommy/Sara/Nobody	
**Blame question**	
If you were Thelma, how much would you blame Tommy for misleading you? • 1 (Not at all)/2/3/4/5/6 (Completely)	

#### 2.1.3 Procedure and design

Experiment 1 used a 2 × 2 between-subject design.^4^ The factors were “incentives” (incentive vs. no-incentive) and “denial” (denial vs. no-denial). Participants were randomly presented with one unique scenario manipulated according to one of four different conditions: incentive and denial, incentive and no-denial, no-incentive and denial, and no-incentive and no-denial.

After reading scenarios, participants responded to two questions: an attention check, which was a multiple-choice question designed to check the reliability of the participant’s answer, and a blame question, which was a 6-point Likert scale question designed to measure the moral reaction of the participant (see Table 1). The attention check was different for each scenario regarding the context while the blame question was the same for every scenario under all conditions; “*If you were listener, how much would you blame the speaker for misleading you?*” [1: Not at all, 2, 3, 4, 5, 6: Completely].

We expected that both the “incentives” and the “denial” factors, as well as their interaction, would cause a significant effect on the moral reaction of the participants. We predicted that participants would blame the speaker in the incentive condition more than in the no-incentive condition, and they would blame the speaker less in the denial condition than in the no-denial condition. However, if the denial was not deemed plausible, as we reasoned would be the case in the incentive condition, we predicted that in the denial condition participants would blame the speaker more, or at least not less, than in the no-denial condition.

### 2.2 Data analysis

In our pre-registered analyses, we planned to use an ordered logistic regression model to test our hypothesis and to include the “scenario” variable as a random factor in case the descriptive statistics showed different distributions of responses across scenarios.^5^ Before the analysis, we thus checked the distributions of participants’ responses and detected such difference across scenarios.^6^ Thus, we added “scenario” as a random factor and switched to multilevel ordered logistic regression model that is a significantly better fit compared to a model with the intercept only, χ*^2^* (4, *N* = 507) = 145.64, *p* < 0.001.

### 2.3 Results

We ran our multilevel ordered logistic regression in Stata 17 (StataCorp, 2021). The results in Table 2 show that speakers’ blameworthiness was modulated by their prior incentives to mislead, but surprisingly not by the denials themselves nor by the interaction between the speakers’ incentives and denials.

**TABLE 2:** Results of the multilevel ordered logistic regression model.

Variable	β	SE (β)	*p*-value	Odds ratio	95% CI of odds ratio	
					Lower bound	Upper bound
**Incentives**						
Yes**	0.620**	**0.229**	**0.007**	**1.858**	**1.185**	**2.912**
No	Reference					
**Denial**						
Yes	0.221	0.223	0.321	1.248	0.806	1.931
No	Reference					
**Incentives × Denial**						
Yes	–0.509	0.320	0.112	0.601	0.302	1.126
No	Reference					

*p* < 0.1, *p < 0.05, **p < 0.01, ***p < 0.001.

Bold values indicate significant variables with *p*-value < 0.05.

The estimated odds ratio of prior incentives points out that participants tended to blame the speakers 1.858 times more (95% CI: 1.185–2.912) by rating higher when the speakers had prior incentives to mislead the audience. However, we could not observe any significant effect of denial on the participants’ rating level.

**FIGURE 1:**
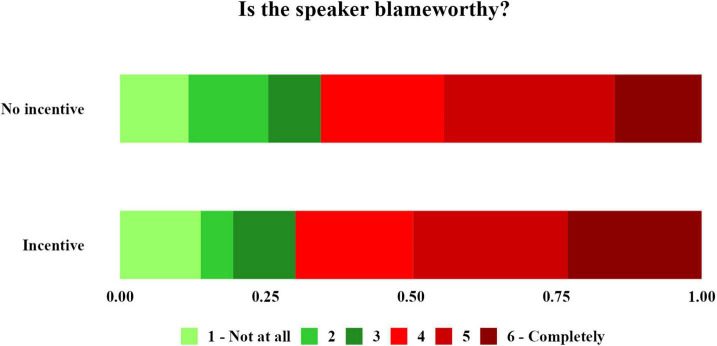
Distribution of participants’ total responses to the blame question. There is a shift towards greater blame in the incentive condition. That is, participants tended to blame the speaker significantly more for misleading the audience when the speaker had prior incentives to do so, independently of the presence of their denial.

Consistent with our prediction, our results reveal that participants’ moral judgements were sensitive to the speaker’s incentives: participants blamed the speakers for a false ascribed meaning significantly more severely when speakers had incentives to do so (see Figure 1). On the other hand, contrary to our prediction, the effects of denial and its interaction with speaker’s incentives were statistically insignificant. The fact that speakers denied the ascribed meaning did not affect the participants’ judgments, regardless of whether the speaker denied an ascribed meaning that they had or had not an incentive to convey in the first place. These findings indicate that speakers, with incentives to mislead their audience, paid higher social costs and were held more blameworthy for misleading their audience, regardless of whether they denied having meant the falsely ascribed meaning.

We assumed that ascribing responsibility to the speaker for having misled the audience, i.e., judging them as blameworthy, is mediated by an interpretation process about speaker’s meaning; and hence that when a denial is offered, the ascription of responsibility should be mediated by a re-interpretation process about speaker’s meaning (as suggested by Mazzarella, 2021). To confirm this, we conducted Experiment 2.

## 3 Experiment 2: Epistemic reaction

A speaker’s denial has an impact on other cognitive processes beyond the ascription of responsibility (see also §4). If an ascribed meaning is plausibly deniable, its denial should first lead to re-interpretation. We designed Experiment 2 to test the hypothesis that the speaker’s prior incentives to mislead the audience are salient contextual assumptions that might block the audience’s re-interpretation of the speaker’s intended meaning.

We measure perceived speaker meaning by asking participants an *interpretation* question. The interpretation question offers two possible interpretations for what the speaker meant (the initially ascribed meaning and the alternative meaning offered with the denial), with degrees of uncertainty. If the participants choose the ascribed meaning over the alternative meaning more frequently when the speaker’s incentives are present than when they are not, this would suggest that the speaker’s prior incentives affect the re-interpretation of what the speaker meant. Additionally, if the participants choose the alternative meaning more often when denial is present, but not when it is absent, that would suggest the denial was plausible. We hence predicted that when the speaker had prior incentives to mislead the audience their denial would not be deemed as plausible, and thus it would not lead to an update of the interpretation of the speaker’s utterance; whereas when the speaker did not have any incentives to mislead their audience, their denial would lead to a re-interpretation of the speaker’s utterance in favour of the alternative proposed.

### 3.1 Methods

The study was pre-registered on OSF.io, with sample size, planned analyses and participants exclusion criteria specified. The pre-registration document is available at https://osf.io/jkn57.

#### 3.1.1 Participants

A statistical power of 95% with α = 0.05 was calculated by the “Basic Functions for Power Analysis (pwr)” package (Champely, 2020) of RStudio 1.4.1103 (R Core Team, 2020) for 752 participants with a small effect size. 790 participants attended the experiment before we closed the experiment and they were recruited *via* Amazon MTurk (Amazon Mechanical Turk^7^). The only criteria for participants was being above 18. Each participant was asked for their consent before starting the experiment and was compensated with $0.40. We excluded data from participants who failed the attention check question (*N* = 38), resulting in 752 participants (360 females, 1 others, 1 prefer not to say, M_age = 40.20); 370 in the incentive condition (121 in the negation and denial condition, 123 in the negation and no-denial condition, 126 in the no-negation condition) and 382 in the no incentive condition (128 in the negation and denial condition, 123 in the negation and no-denial condition, 131 in the no-negation condition).

#### 3.1.2 Materials

To maintain comparability with the previous study, we used the same four scenarios that were used in Experiment 1. However, contrary to Experiment 1, we additionally manipulated the negation part in order to isolate the effect of denial on re-interpretation. The scenarios followed the structure below.

• *Context part* depicted a social situation between a speaker and a listener, and included information about the speaker’s incentives to mislead the listener.
• *Dialogue part* included a question of the listener about an event; the speaker’s response to the question was an utterance (X) which yielded an ascribed meaning (Y). The dialogue part was identical in all conditions.
• *Negation part* included the information about the listener realising that Y is false. The evidence that Y is false was present in the negation condition and absent in the no-negation condition.
• *Denial part* included the dialogue in which the listener confronts the speaker by stating that “I thought you said Y” and the speaker denied having meant that Y (ascribed meaning), and offered an alternative meaning (Z) for their utterance X: “*I didn’t say that. I just meant Z*”. As in Experiment 1, the denial part was present only in the denial condition.

Table 3 shows an example. Again, all stimuli are available at https://osf.io/bmqk4/.

**TABLE 3:** Example of the scenario structure and measures used in the Experiment 2.

Context–incentive	Context–no-incentive
Tommy and Thelma have been in a relationship for a few years.	Tommy and Thelma are siblings and have a very close relationship.
They live in the same college dorm. At the beginning of the new term, they meet a new student, Sara, in the dorm cafeteria. Tommy and Sara start spending a lot of time together, and Tommy knows that Thelma also likes Sara and is happy about them hanging out. One day Thelma looks for Tommy and cannot find him anywhere.	
**Dialogue** Thelma asks Tommy when he is back: *“Where were you? I couldn’t find you anywhere.”* Tommy answers: *“Sorry, I went to the laundry room.”*	
**Negation**	**No-negation**
Later, some friends tell Thelma that Tommy and Sara were together that afternoon.	**x**
**Denial**	**No-denial**
Thelma says to Tommy: *“I thought you said that you were doing your laundry.”* Tommy answers: *“Oh no, I didn’t say that. I just meant that I was helping Sara because she didn’t know how to use the washing machine and asked me for help.”*	**X**
**Attention check**	
Who was Thelma looking for that afternoon? • Tommy/Sara/Nobody	
**Interpretation question**	
When Tommy said “I went to the laundry room”, did Tommy mean he was doing his laundry or he was helping Sara? ❖ Tommy clearly meant he was doing his laundry ❖ Tommy probably meant he was doing his laundry ❖ what Tommy meant is unclear ❖ Tommy probably meant he was helping Sara ❖ Tommy clearly meant he was helping Sara	

Bold values indicate significant variables with *p*-value < 0.05.

#### 3.1.3 Procedure and design

Experiment 2 used a 3 × 2 between-subjects design. The factors were “incentives” (incentive vs. no incentive), “denial” (denial and no-denial), and “negation” (negation and no-negation). Participants were randomly presented with one unique scenario manipulated according to one of six different conditions: incentive and denial, no-incentive and denial, incentive and negation, no-incentive and negation, incentive and no-negation, no-incentive and no-negation.

After reading the scenario, participants responded to two questions: the attention check, and the interpretation question, which was a multiple-choice question with five independent levels and designed to check which meaning is understood to be conveyed by the speaker, the ascribed or the alternative meaning, and with how much certainty. The interpretation question was the same for every scenario under all conditions: “*When [speaker] said utterance (X), did [speaker] mean ascribed meaning (Y) or alternative meaning (Z)?*” [the speaker clearly meant the ascribed meaning (Y); the speaker probably meant the ascribed meaning (Y); what the speaker meant is unclear; the speaker probably meant the alternative meaning (Z); the speaker clearly meant the alternative meaning (Z)].

We hypothesised that the speaker’s incentives to mislead the audience would impact the plausibility of their denial, thus we expected an interaction between the “denial” and the “incentives” factors. We further expected that the “negation” factor may have an additional significant effect alone on participants’ responses without the presence of the speaker’s denial. We thus predicted:

• A significant effect of “incentives”: participants would choose the alternative meaning more often in the no-incentive conditions than in the incentive conditions.
• An interaction between “denial” and “incentives” factors: participants would choose the alternative meaning more often in the denial condition than in the no denial condition, but only in the no-incentive conditions.
• A significant effect of “negation”: participants would choose the alternative meaning more often in the negation conditions than in the no-negation condition.

### 3.2 Data analysis

To test our hypothesis, we pre-registered that we will use a multinomial logistic regression model to analyse a categorical dependent variable, i.e., participants’ responses to interpretation question in our model, with more independent factors, i.e., “incentives”, “negation” and “denial” factors.^7^ We ran two separate models with the same dependent but different independent variables for the ease of the analysis: (1) a denial model that included the “incentives” factor, the “denial” factor, and their interaction, (2) a negation model that included the ‘incentives’ factor, the ‘negation’ factor, and their interaction. In both of our models, we chose the “speaker clearly meant the ascribed meaning” level of dependent variable as our base category value. Both of our models improved their fit when we added the ‘scenario’ as a random effect and switched to multilevel multinomial logistic regression, denial model, χ*^2^* (1, *N* = 506) = 5.28, *p* = 0.022, and negation model, χ*^2^* (1, *N* = 503) = 13.30, *p* = 0.001. Additionally, both of our models fit significantly better compared to model with the intercept only; denial model, χ*^2^* (13, *N* = 506) = 92.74, *p* < 0.001, and negation model, χ*^2^* (13, *N* = 503) = 79.03, *p* < 0.001.

### 3.3 Results

We ran both of our multilevel multinomial logistic regression models in Stata 17 (StataCorp, 2021). The results of both models are shown in Table 4.

**TABLE 4:** Effects of the “incentives”, “denial” and “negation” factors on participants’ responses to the interpretation question in the multilevel multinomial regression denial model and negation model.

Model	Effect	Model fitting criteria	Likelihood ratio tests		
		−2 Log likelihood of reduced model	Chi-squared	Degrees of freedom	*p*-value
Denial model	Intercept	1522.12	0.000	0	–
	Incentives	1519.95	2.17	4	0.705
	Denial***	**1484.06**	**38.06**	**4**	**0.000**
	Incentives*Denial	1521.24	0.88	4	0.927
Negation model	Intercept	1494.96	0.000	0	–
	Incentives	1492.79	2.17	4	0.704
	Negation***	**1466.37**	**28.59**	**4**	**0.000**
	Incentives*Negation	1491.65	3.31	4	0.507

*p* < 0.1, *p < 0.05, **p < 0.01, ***p < 0.001.

Bold values indicate significant variables with *p*-value < 0.05.

Contrary to our prediction, the speaker’s incentives to mislead the audience did not affect participants’ interpretation of the intended meaning overall. No significant effect of “incentives” was found. However, the presence of denial did have a significant effect on the participants’ responses. This suggests that participants are disposed to think that they misinterpreted the intended meaning and to accept the alternative as the originally intended meaning. Thus, the denial model did not confirm our prediction regarding the effect of the speaker’s incentives on their re-interpretation process. Our results suggest that, as proposed by Mazzarella (2021), the presence of a speaker’s denial triggers a re-interpretation process.

**FIGURE 2:**
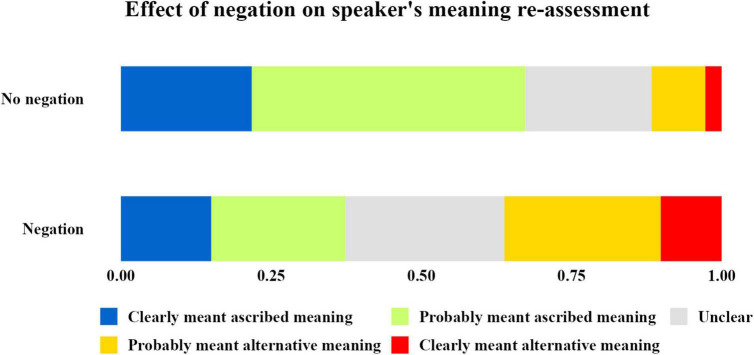
Distribution of participants’ total responses to the interpretation question, as the measure of effect of the “negation” factor. As we can see, when provided with the information about the ascribed meaning being false, participants were significantly more likely to re-interpret speaker meaning.

Also, as we predicted, when participants were provided with the information that the ascribed meaning was false, this affected participants’ assessments of the intended meaning (see Figure 2). The new information caused participants to update their belief about what the speaker intended to communicate, even when they were not provided with the speaker’s denial of the ascribed meaning.

Collectively, these results show that people are able and willing to retrospectively ascribe a different informative intention to the speaker. When presented with relevant information such as a denial or a negation of their ascribed meaning, the re-interpretation process occurs. Perhaps surprisingly, we did not find evidence that the interpretation and re-interpretation processes are sensitive to the speaker’s incentives to mislead the audience; participants were rather inclined to revise their interpretation in both situations.

## 4 “That’s not what i meant!” rethinking deniability

Our findings show that a satisfactory account of plausible deniability relies on disentangling multiple facets of audiences’ reactions to denials. In particular, the dissociation we observed between moral (§2) and epistemic (§3) reactions towards denials was unpredicted and is puzzling. While participants’ moral reaction have been found to be influenced by the speaker’s incentives to mislead the audience, but not by their (the speaker’s) denial, the opposite was found for participants’ epistemic reactions; that is, participants’ reported willingness to re-interpret speaker meaning was influenced by their (the speaker’s) denials, and other evidence that the initially ascribed meaning was false, but not by their incentives to mislead. In light of these findings, here we re-analyse deniability, making distinctions that have not been clearly made in the previous literature on the topic. These distinctions are inspired in particular by the Relevance Theory approach to communication and cognition (e.g., Wilson and Sperber, 2012; Mazzarella, 2021; Heintz and Scott-Phillips, 2022), but could also be derived from other theoretical frameworks.

It is essential to distinguish a communicator’s intended meaning from what could be called the “ascribed” meaning; that is, the meaning the audience ascribes to the utterance. Denials are statements from the communicator about how the meaning ascribed by the audience differs from the communicator’s intended meaning. Such mismatches between intended meaning and ascribed meaning occur all the time in ordinary communication, and humans have developed and use a wide array of mechanisms for “repairing” dialogue when this occurs (Dingemanse and Enfield, 2015; Dingemanse et al., 2015). These include interjections such as “Huh?” and “What?”; question words seeking clarification; partial repeats of the source of uncertainty followed by a question word; reformulations of what was meant; and others. However, some of the time, denials trigger further cognitive reactions in audiences that go beyond repair, and corresponding clarification of what the communicator had originally meant.

At least three possible reactions should be distinguished. These are not mutually exclusive, and will in some cases co-occur with one another.

*a. Audiences may re-interpret the original utterance, potentially in line with the new interpretation offered by the speaker.*

Denials often are accompanied by an alternative interpretation aimed to trigger a re-interpretation process in the audience. For instance, in order to deny to have lied with his infamous statement “*I did not have sexual relations with that woman*”, former US president Bill Clinton offered the alternative interpretation for which the definition of “sexual relations” was thought not to include the specific interactions he admittedly had with Lewinsky. Reinterpretations typically require increasing the saliency of some contextual assumptions that were neglected when the communicative act was initially produced. If the new alternative interpretation meets a better trade-off in terms of cognitive utility compared to the old interpretation, then the re-interpretation successfully occurs and the denial can be perceived as plausible (Mazzarella, 2021). In our Experiment 2, participants reported a willingness to re-interpret the communicator’s original utterances in this way. The output of this process can be described as a type of intuitive belief, because it consists in inferences that are spontaneous, implemented by our communicative capacities. Specifically, the belief about re-interpretation is not based on an assessment of the reasons for forming such belief (on the difference between intuitive and reflective beliefs, see Sperber, 1997; Mercier and Sperber, 2019).

*b. Audiences may ascribe responsibility to speakers for the cognitive effects caused by their earlier communicative act, especially when the audience had relied on those cognitive effects.*

In general, speakers are held accountable for the cognitive effects caused by their communicative acts, rather than being responsible for, more narrowly, only the effects of what was “literally” said (Morency et al., 2008; Haugh, 2013; Bonalumi et al., 2020; Yuan and Lyu, 2022). These accountability effects may be particularly sensitive to the plausibility of the denial. The fact that the speaker suggests that there is a mismatch between the intended meaning and the ascribed meaning may mitigate blameworthiness, but can also backfire if the denial is not plausible, and even more so when speakers deny having intended these cognitive effects (Bonalumi et al., 2022; see also Oswald, 2022, for a similar take on insinuations). Bill Clinton’s denial attempt certainly was not convincing and triggered additional public outrage. When speakers attempt to eschew the responsibility for the cognitive effects they had generated in the audience, then the audience’s moral evaluation of the speaker is impacted.

*c. Audiences may reflexively accept or reject a denial, considering the evidence they possess that the speaker really meant the ascribed meaning. These reflective beliefs include anticipating an argument with the speaker about what he or she truly intended.*

The audience’s confidence in explicitly or publicly attributing an informative intention to the speaker is informed not only by their intuitive interpretation of the utterance, but also by considerations of the evidence in favour of attributing to the speaker the ascribed meaning, i.e., the denied meaning, or the alternative meaning. The belief about the speaker’s actual intended meaning is, in that case, a reflective belief. In fact, the audience may reflect that meanings that are “literally” expressed as less deniable than meanings that are implied: this is because the uttered words can easily be used as good evidence for ascribing literal meanings. By contrast, implied meanings are (intuitively) attributed on numerous contextual cues that might be harder to use as evidence in an argument about intended meaning. More generally, reasoning on ascribed meaning is likely to ensure that denials are accepted more often than not because of the widely shared intuitions that one has a privileged access to one own’s thoughts and intentions, and thus it is not possible to really know what others think (Keane, 2008; Astuti, 2015; see also Burge and Peacocke, 1996)—or that language is a digital medium that encodes meaning (Pinker, 2004). The reflexive acceptation or rejection of denial thus involve other considerations such as higher-order deniability, possible deniability, and relationship management concerns (Lee and Pinker, 2010; Dinges and Zakkou, in press; see also Elder, 2021, for a discussion on deniability and micro-aggression). Additionally, the audience’s confidence in the ascribed meaning can be informed by further contingent elements that suggest that engaging in an overt reproach of the speaker will or will not be successful (e.g., power relations, appropriateness of the reproach, etc.) (see also Dinges and Zakkou, in press). In particular, both speaker’s reputation and their institutional roles are factors that we did not manipulate here, but we expect them to affect significantly the perceived plausibility of a denial and as such they should be explored in future research.

These three possible cognitive reactions can be present simultaneously. Consider, for example, the famous yacht scene from “The Wolf of Wall Street”^8^: Jordan Belford (Leonardo DiCaprio) implicitly attempts to bribe an FBI agent (Kyle Chandler) by stating “*I’d do that [providing information about a millionaire stock trade] for anybody who needs a proper guidance.*” Once confronted by the FBI agent, (“*You just tried to bribe a federal officer.*”), Belford denies having had such intention (“*I don’t know what you’re talking about.*”). The FBI agent appears to hold the intuitive belief that Jordan Belford’s denial is implausible, maintaining the inferred informative intention of a bribe proposal (“*C’mon you know what I’m talking about.*”), but at the same time may lack the confidence to publicly attributing this informative intention following Jordan’s denial, in particular in front of third parties—and indeed the immediate accusation of bribe is not acted upon (as Belford points out, “*That would not hold up in a court of law.*”). During the whole interaction, in any case, the FBI agent is clearly taking a moral stance against the broker (e.g., “*You, Jordan, you got this way all on your own—Good for you little man*.”), which is of course unaffected by his (Belford)’s denial attempt.

Existing experimental work, including our own, does not adequately control for judgements informed by reflective beliefs about possible deniability, i.e., possibility (c) above. In the two studies we have presented here, we investigated the effect of speaker’s denials and speaker’s incentives to mislead the audience on moral and epistemic evaluations. As we reported in §2–3, our results are only partially in line with these predictions. We suggest this may be because our experimental designs have conflated reaction (c) with either of the other two. Our intention was to target reactions (a), intuitive reinterpretation, and (b), responsibility ascription. However, we may have additionally triggered reaction (c), reflective beliefs, in particular argumentative considerations and participants’ judgements about possible deniability instead.

Partly because we expected that epistemic and moral reactions would be consistent, we reason that the mismatch that we found between the reported re-interpretations and the ascriptions of responsibility may be due to the interference of such reflective beliefs. More specifically, we suggest that participants’ reported epistemic judgements (a) could have been conflated with other argumentative considerations (c). Such argumentative considerations would have prevented participants from engaging in an explicit accusation about speaker’s intentions. However, these considerations may have had less impact on participants’ moral judgements (b); or they may even have been consistent with such judgements. In fact, and regardless of their actual intention to mislead, the favourable outcome for the speaker (i.e., their incentives) is good enough evidence for defending an explicit disapproval of the speaker’s incompetent behaviour.

The difficulty of keeping these different reactions apart in experimental design is one that may have recurred in other recent research on deniability (Sternau et al., 2015, 2017; Bonalumi et al., 2022). Future experimental designs must focus on operationalising plausible deniability in a way that tears apart (c) from (a) and (b). A deeper understanding of the diverse range of ways in which people react to denials will be hindered unless and until this methodological problem is resolved.

The important general point is that plausible deniability involves strategic cognition for both speakers and audiences. The speaker attempts not to produce evidence to be accused of lying, while the audience assesses whether the speaker has or had the intention to mislead. Thus, while audience may modulate their (re-)interpretation of what is said in view of the speaker’s intentions, discussing a denial involves not only re-interpretation of the speaker’s informative intention, but engagement in discussion or argument about those intentions.; and since speakers can always claim privileged access to their own past intentions, the audience may strategically avoid this outcome. A good cognitive description of plausible deniability must account for these different processes.

## Data availability statement

The datasets presented in this study can be found in online repositories. The names of the repository/repositories and accession number(s) can be found below: https://osf.io/jkn57.

## Ethics statement

The studies involving human participants were reviewed and approved by United Ethical Review Committee for Research in Psychology (EPKEB). The patients/participants provided their written informed consent to participate in this study.

## Author contributions

FB: conceptualization, methodology, validation, data curation, writing – original draft, writing – review and editing, visualization, and project administration. FBB: software, validation, formal analysis, investigation, data curation, writing – original draft, visualization, and funding acquisition. TS-P: validation, writing – original draft, and writing – review and editing. CH: conceptualization, resources, writing – review and editing, supervision, and funding acquisition. All authors contributed to the article and approved the submitted version.

## Footnotes

1 The examples are respectively taken from scenes from the movies The Wolf of Wall Street (Scorsese, 2013), in which the broker Belford suggests that he could involve the FBI agent into a 500mln trade; North by Northwest (Hitchcock, 1959), in which Eve invites Roger to sleep together in her train couchette; and Game of Thrones (Benioff and Weiss, 2011), in which Ned Stark insinuates that Jamie Lannister is a coward.

2 There is a sizeable literature on whether conveying indirectly false content should qualify as lying rather than merely misleading, both theoretical (e.g., Carson, 2006; Meibauer, 2014, 2018; Viebahn, 2017; Saul, 2018; Marsili, 2021), and experimental (e.g., Danziger, 2010; Wiegmann et al., 2016, 2021; Willemsen and Wiegmann, 2017; Antomo et al., 2018; Reins and Wiegmann, 2021; Viebahn et al., 2021). For this study we do not take any particular theoretical stance on what counts as lie; although people may entertain the belief that lying is morally worse than misleading (see Chisholm and Feehan, 1977; Adler, 1997), if only for argumentative reasons (see § 4).

3
https://www.mturk.com

4 We opted for a between-subject design because we reasoned that a within-subject design could trigger an experimenter effect if participants were offered both conditions of the same scenario.

5 see https://osf.io/bmqk4/

6 Our model fits the data better compared to the same model without random effect, χ*^2^* (1, *N* = 507) = 140.04, *p* < 0.001, and improves the significance level and estimated odds ratio of our already significant variables as results show in Table 2. See https://osf.io/bmqk4/for more details.

7 In pre-registration, we mentioned a gender effect that was observed while piloting the study. When, we added the “gender” variable to both of our models, we did not detect any significant gender effect (in the denial model, χ*^2^* (4, *N* = 506) = 3.49, *p* = 0.480, 2); in the negation model, χ*^2^* (4, *N* = 503) = 0.16, *p* = 0.997). Also, we did not have any pre-assumption on why gender should have an effect, so we excluded ‘gender’ from our models.

8 See https://www.youtube.com/watch?vojWbip26nQs
